# How to reduce the risk of cervicalgia and low back pain in obese individuals: A mendelian randomization study

**DOI:** 10.1097/MD.0000000000033710

**Published:** 2023-05-05

**Authors:** Li Gou, Qiang Zheng

**Affiliations:** a Operation Room, Sichuan Academy of Medical Sciences & Sichuan Provincial People’s Hospital, Chengdu, China; b Emergency Department, The First Hospital of Jilin University, Changchun, China.

**Keywords:** cervicalgia, low back pain, mediation analyses, mendelian randomization, obesity

## Abstract

Obesity is associated with cervicalgia and low back pain (LBP), but the specific role and how to reduce the risk of neck pain and low back pain are not clear. The Mendelian randomization analysis was used to investigate the causal relationship between obesity and cervicalgia and LBP, as well as the effect of possible mediating factors. Then, causal associations were estimated using sensitivity analysis. Educational level (odds ratio (OR) = 0.30, 0.23) was negatively associated with cervicalgia and LBP; Heavy physical work (HPW) (OR = 3.24, 2.18), major depression (MD) (OR = 1.47, 1.32), body mass index (BMI) (OR = 1.36, 1.32), and waist circumference (WC) (OR = 1.32, 1.35) were positively associated with cervicalgia and LBP; Leisure sedentary behavior (LSB) (OR = 1.96), smoking (OR = 1.32), and alcohol intake frequency (OR = 1.34) were positively associated only with LBP, but not with cervicalgia. Ranked by mediated proportions of selected mediators, the largest causal mediator from BMI and WC to cervicalgia was educational level (38.20%, 38.20%), followed by HPW (22.90%, 24.70%), and MD (9.20%, 17.90%); However, the largest causal mediator from BMI and WC to LBP was LSB (55.10%, 50.10%), followed by educational level (46.40%, 40.20%), HPW (28.30%, 20.90%), smoking initiation (26.60%, 32.30%), alcohol intake frequency (20.40%, 6.90%), and MD (10.00%, 11.40%). For obese individuals, avoiding HPW and maintaining a stable mood may be an effective approach to prevent cervicalgia; Additionally, reducing LSB, avoiding HPW, quitting smoking and drinking, and maintaining a stable mood may be an effective approach to prevent LBP.

## 1. Introduction

Obesity is associated with multiple musculoskeletal disorders including cervicalgia and low back pain (LBP) and thus reduced the motility and life quality of the patients.^[1,2]^

Cervicalgia and LBP are major public health concern worldwide.^[3–5]^ According to the World Health Organization report concerning “the world’s top 10 intractable diseases,” cervicalgia and LBP ranked second, in which among nearly 8 billion people in the world, there are within 2 billion cervicalgia and LBP patients, indicating that it is one of the most common diseases. Cervicalgia and LBP are engulfing more and more people’s lives and health, and also imposing a heavy burden to the global economy. In 2016, cervicalgia and LBP were the disease with the highest healthcare expenditure in the United States, out of 154 conditions, with an estimated $134.5 billion.^[6]^ In 2012, 25.5 million Americans missed their work due to cervicalgia and LBP, with an average of 11.4 days. In 2017, the global age-standardized prevalence and incidence of cervicalgia and LBP were 3551.1/100,000 and 806.6/100,000, respectively.^[7]^ There is no definitive treatment for cervicalgia and LBP, thus, it is important to identify risk factors for prevention and early diagnosis of cervicalgia and LBP.^[8]^ Weight loss in obese individuals can significantly reduce the risk of cervicalgia and LBP, but it is very difficult. Therefore, it is important to find some methods to significantly reduce the risk of cervicalgia and LBP in obese individuals.

Mendelian randomization (MR) method has been widely used in the causality research of genome-wide association study (GWAS) data in recent years. It is used to characterize random division and combination during the formation of genetic variation gametes to regroup the population randomly, which theoretically avoids the influences of confounding factors. In addition, the differences explained by genetic variations (exposed instrumental variables) have priority over outcome-based explanations, thereby eliminating the reverse causality issue.^[9–11]^ This phenomenon has become a research hotspot in recent years. The present study used 2-sample MR method and single-nucleotide polymorphisms (SNPs) as a tool, and analyzed the causal association between obesity and cervicalgia and LBP, as well as the effect of possible mediating factors at the genetic level based on GWAS data.

## 2. Methods

### 2.1. Study design

**Figure 1.**
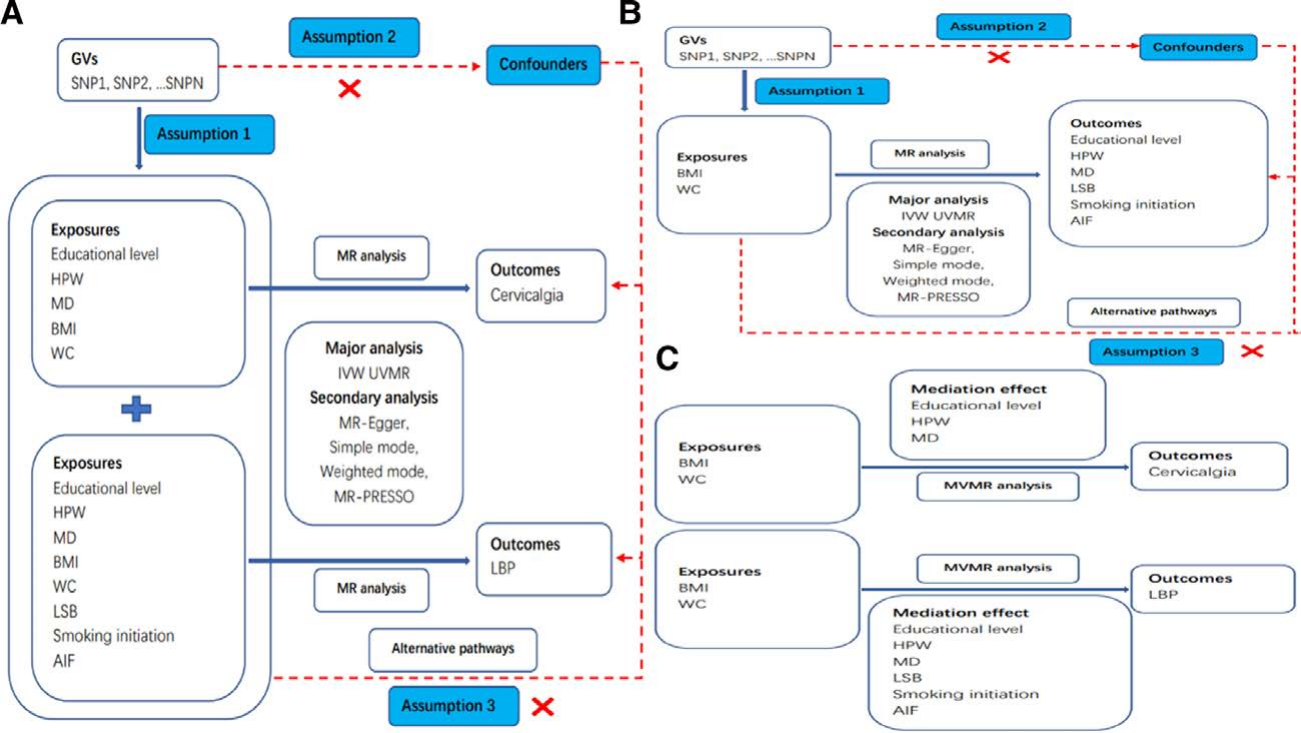
An overview of the analytical plan. AIF = alcohol intake frequency, BMI = body mass index, HPW = heavy physical work, LSB = leisure sedentary behavior, LBP = low back pain, MD = major depression, WC = waist circumference.

This study included 3 stages of analyses (for study design see Fig. 1). In stage 1, we assessed the causal associations of educational level, heavy physical work (HPW), body mass index (BMI), waist circumference (WC) and major depression (MD) with cervicalgia using univariate MR (UVMR) (Fig. 1A). we assessed the causal associations of educational level, HPW, BMI, WC, MD, leisure sedentary behavior (LSB) (proxied with television watching), smoking initiation and alcohol intake frequency (AIF) with LBP using UVMR (Fig. 1A). Next, in stage 2, we assessed the causal associations of BMI and WC with educational level, HPW, MD, LSB, smoking initiation and AIF using UVMR (Fig. 1B). Finally, in stage 3, we identified candidate mediators of the association between obesity and cervicalgia and LBP and calculated their mediating effects using 2-step MR (Fig. 1C). Moreover, GWAS data were used for analysis in accordance with the STROBE-MR guidelines.^[12]^ All study participants provided written informed consent.

### 2.2. MR and assumptions

This is a 2-sample MR study. Genetic tools, including SNPs were used to predict the causality of exposure and outcome from the latest GWAS. MR requires the instrumental variable (IV) to meet 3 assumptions: IV should be associated with exposure; IV should not be associated with confounding factors, while it should be associated with exposure and outcome; The effect of IV on the results is achieved through exposure.^[13]^

### 2.3. Data sources

#### 2.3.1. Exposure

The datasets used in this analysis are publicly available. The latest exposure data were downloaded from the IEU Open GWAS project (https://gwas.mrcieu.ac.uk/) and UK biobank (https://www.ukbiobank.ac.uk/). Risk factors for exposure included Qualifications: College or University degree, Job involves heavy manual or physical work, MD, BMI, WC, Time spent watching television, Smoking initiation, and AIF.

Furthermore, GWAS with large sample sizes and cohorts consisting exclusively of individuals from European ancestry were used as data sources for phenotypic genetic analysis. First, SNP associated with each selected trait at a genome-wide significance threshold of *P* < 5E-8 was included. Linkage disequilibrium was estimated among SNPs based on the 1000 Genomes European Reference panel.^[14]^ The clump program in PLINK software was used to exclude the dependent variables with r^2^ < 0.001. Besides, r^2^ was calculated using the 1000 Genomes European Reference panel. Then, in order to quantify the intensity of SNPs, the F statistic of SNPs of each group was calculated. Detailed information on GWASs of exposures and outcome is presented in Table 1.

**Table 1:** Detailed information on GWASs of exposures and outcome.

Exposure or outcome	IVs	IVs available	Cochran Q	Q–*P* value	Intercept	*P* for intercept	Outliers	Sample size	Ancestry	Units	SNPs	F
Exposure												
Educational level	261	253/238	249	.267	−0.004	0.635	0	458,079	European	SD	9,851,867	10
HPW	25	22/21	14	.790	0.019	0.626	0	263,615	European	SD	9,851,867	29
BMI	458	442/415	453	.087	0.001	0.849	0	461,460	European	SD	9,851,867	61
WC	374	360/339	333	.558	−0.004	0.334	0	462,166	European	SD	9,851,867	44
MD	50	46/44	34	.789	0.001	0.960	0	500,199	European	NA	NA	183
Outcome												
Cervicalgia	-	-	-	-	-	-	-	167,956	European	Odds ratio	16,380,284	-
Exposure												
Educational level	261	253/233	251	.180	0.001	0.736	0	458,079	European	SD	9,851,867	10
HPW	25	22/21	30	.057	0.021	0.396	0	263,615	European	SD	9,851,867	28
BMI	458	442/402	445	.058	0.001	0.861	0	461,460	European	SD	9,851,867	60
WC	374	360/328	355	.126	−0.002	0.341	0	462,166	European	SD	9,851,867	43
MD	50	45/40	50	.096	0.026	0.119	0	500,199	European	NA	NA	173
LSB	113	108/99	115	.105	−0.016	0.060	0	437,887	European	SD	9,851,867	22
Smoking initiation	93	90/79	94	.093	−0.009	0.325	0	607,291	European	NA	11,802,365	161
AIF	99	97/92	113	.051	−0.002	0.739	0	462,346	European	SD	9,851,867	117
Outcome												
LBP	-	-	-	-	-	-	-	177,860	European	Odds ratio	16,380,287	-

AIF = alcohol intake frequency, BMI = body mass index, GWAS = genome-wide association study, HPW = heavy physical work, IVs = instrumental variables, LBP = low back pain, LSB = leisure sedentary behavior, MD = major depression, SD = standard deviation, SNPs = single-nucleotide polymorphisms, WC = waist circumference.

#### 2.3.2. Outcome

In the present study, GWAS summary statistics for cervicalgia and LBP were obtained from the FinnGen cohort (https://www.finngen.fi/en). Including 3274 patients with cervicalgia and 164,682 controls up to 2021. It was attempted to analyze GWAS summary statistics from the FinnGen cohort using 16,380,284 SNPs. Including 13,178 patients with LBP and 164,682 controls up to 2021. It was attempted to analyze GWAS summary statistics from the FinnGen cohort using 16,380,287 SNPs.

### 2.4. Statistical analysis

Inverse-variance weighting (IVW) is the primary method in MR. When its assumption is true, IVW is the most potent method for MR. However, if some tools violate the IV hypothesis, the MR analysis may present wrong results^[15]^ (if it is empty, it indicates a causal association). Next, sensitivity analysis was conducted. First, *Q* statistic was used to quantify the relative goodness of fit of MR-Egger over the IVW approach.^[16]^ Second, MR-Egger was utilized to estimate the level of pleiotropy according to its interception to ensure that genetic variation is independently associated with exposure and results.^[16]^ The stability and robustness of the results were elevated by additional analysis (weighted median method^[17]^ and weighted mode method^[18]^) of MR with different modeling assumptions and advantages. The weighted median method combines data from multiple genetic variants into a single causal estimate. This method is consistent with estimators even when up to 50% of the information comes from invalid instrumental variables. The weighted mode approach can perform MR robustly and efficiently in the presence of invalid IVs. Compared with other robust methods, it has the lowest mean square error in a series of real data.^[18]^ Third, MRPRESSO was utilized to detect the outliers and correct the pleiotropy.^[19]^ Fourth, SNP and retention analysis were used to evaluate the possible association driven by a SNP. Scatter and funnel plots were drawn to rule out the potential outliers. Finally, SNPs associated with any exposure were searched using the *phenoscanner* package of R software, in which the identified pleiotropic SNPs were excluded,^[20]^ and MR IVW was reperformed to examine the robustness of causal effects. Then, multivariate MR analysis and mediation MR analysis were performed.

### 2.5. Mediation MR analysis

For the mediation MR analysis, we performed conventional network MR.^[21]^ Specifically, we first estimated the effect of BMI/WC on mediators (Educational level, HPW, MD, LSB, Smoking initiation, and AIF) using the IVW MR approach. Next, we applied regression-based multivariable MR to estimate the effect of mediators respectively on risk of cervicalgia and LBP, adjusting for the genetic effect of the instruments on BMI or WC respectively.^[22]^ The indirect effect of the considered exposure on cervicalgia and LBP risk mediated through mediators was estimated by multiplying results from these 2 MR analyses. We finally divided the mediated effect by the total effect to estimate the proportion mediated, as previously done.^[21]^

Statistical analysis was conducted using R 4.2.2 software (https://posit.co/products/open-sources/rstudio). MR software package was used for MR analysis,^[23]^ and MRPRESSO adopted MRPRESSO package.^[23]^

## 3. Results

Table 1 and Table 2 summarize the GWAS of SNP traits used as genetic tool variables and the results of sensitivity analysis; Further details are provided in Table S1, Supplemental Digital Content, http://links.lww.com/MD/I937 and Table S2, Supplemental Digital Content, http://links.lww.com/MD/I938.

**Table 2:** Detailed information on GWASs of exposures and outcome.

Exposure	Outcome	IVs	Cochran Q	Q–*P* value	Intercept	*P* for intercept	Outliers	F
BMI	Educational level	257	292	.055	−0.001	0.057	2	57
	HPW	337	339	.426	0.001	0.258	1	57
	MD	309	342	.083	−0.001	0.523	0	61
	LSB	242	254	.253	0.001	0.288	1	55
	Smoking initiation	280	317	.054	−0.001	0.066	0	62
	AIF	270	302	.075	0.001	0.448	2	50
WC	Educational level	195	224	.062	−0.001	0.591	2	40
	HPW	291	322	.088	0.001	0.234	1	43
	MD	247	279	.068	−0.001	0.764	0	40
	LSB	216	247	.060	0.001	0.111	1	39
	Smoking initiation	203	211	.298	−0.002	0.067	0	36
	AIF	223	251	.079	−0.001	0.724	1	37

AIF = alcohol intake frequency, BMI = body mass index, GWAS = genome-wide association study, HPW = heavy physical work, IVs = instrumental variables, LSB = leisure sedentary behavior, MD = major depression, WC = waist circumference.

### 3.1. Effects of modifiable risk factors on cervicalgia and LBP

**Figure 2.**
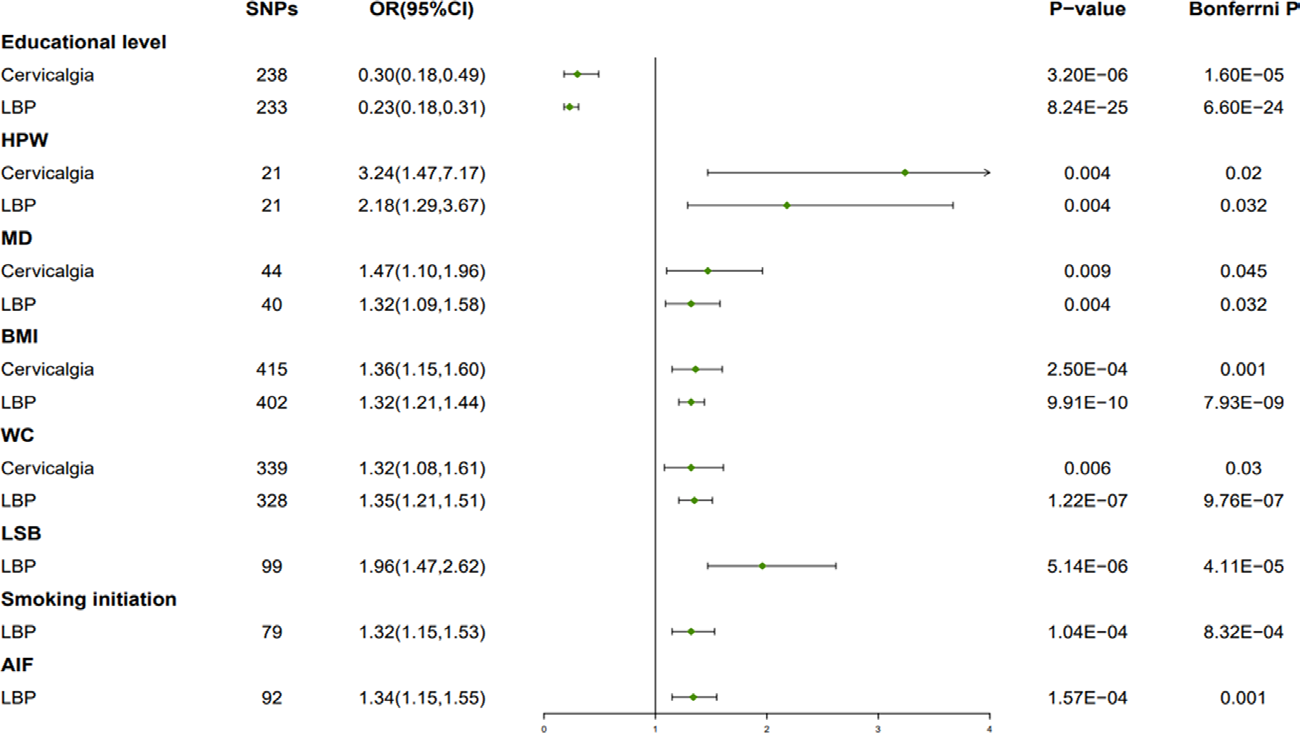
MR Analysis of BMI, WC, and mediating factors with cervicalgia and LBP. AIF = alcohol intake frequency, BMI = body mass index, HPW = heavy physical work, LSB = leisure sedentary behavior, LBP = low back pain, MD = major depression, MR = Mendelian randomization, WC = waist circumference.

In the UVMR analysis of the associated risk factors, it was found that the following modifiable risk factors had a significant causal effect on cervicalgia and LBP (Fig. 2 and Table S1, Supplemental Digital Content, http://links.lww.com/MD/I937). For every 1 unit increase in educational level, relative risk of cervicalgia decreased by 70.0% (odds ratio (OR) = 0.30, 95% confidence interval (CI): 0.18–0.49, *P* = 3.20E-06, Bonferrni *P* = 1.60E-05) and LBP decreased by 77.0% (OR = 0.23, 95% CI: 0.18–0.31, *P* = 8.24E-25, Bonferrni *P* = 6.60E-24). For every 1 unit increase in HPW, relative risk of cervicalgia increased by 224.0% (OR = 3.24, 95% CI: 1.47–7.17, *P* = .004, Bonferrni *P* = .02) and LBP increased by 118.0% (OR = 2.18, 95% CI: 1.29–3.67, *P* = .004, Bonferrni *P* = .032). For every 1 unit increase in MD, relative risk of cervicalgia increased by 47.0% (OR = 1.47, 95% CI: 1.10–1.96, *P* = .009, Bonferrni *P* = .045) and LBP increased by 32.0% (OR = 1.32, 95% CI: 1.09–1.58, *P* = .004, Bonferrni *P* = .032). For every 1 unit increase in BMI, relative risk of cervicalgia increased by 36.0% (OR = 1.36, 95% CI: 1.15–1.60, *P* = 2.50E − 04, Bonferrni *P* = .001) and LBP increased by 32.0% (OR = 1.32, 95% CI: 1.21–1.44, *P* = 9.91E − 10, Bonferrni *P* = 7.93E − 09). For every 1 unit increase in WC, relative risk of cervicalgia increased by 32.0% (OR = 1.32, 95% CI: 1.08–1.61, *P* = .006, Bonferrni *P* = .03) and LBP increased by 35.0% (OR = 1.35, 95% CI: 1.21–1.51, *P* = 1.22E − 07, Bonferrni *P* = 9.76E − 07). For every 1 unit increase in LSB, relative risk of LBP increased by 96.0% (OR = 1.96, 95% CI: 1.47–2.62, *P* = 5.14E − 06, Bonferrni *P* = 4.11E − 05). For every 1 unit increase in Smoking initiation, relative risk of LBP increased by 32.0% (OR = 1.32, 95% CI: 1.15–1.53, *P* = 1.04E − 04, Bonferrni *P* = 8.32E − 04). For every 1 unit increase in AIF, relative risk of LBP increased by 34.0% (OR = 1.34, 95% CI: 1.15–1.55, *P* = 1.57E − 04, Bonferrni *P* = .001).

Educational level was negatively associated with cervicalgia and LBP; HPW, MD, BMI, and WC were positively associated with cervicalgia and LBP; LSB, smoking initiation, and AIF were positively associated only with LBP, but not with cervicalgia (Fig. 2). The results of the above-mentioned MR analysis were robust to several sensitivity analyses (Table 1 and Table S1, Supplemental Digital Content, http://links.lww.com/MD/I937). All F statistics exceeded 10 for all instruments, indicating satisfactory strength of the genetic instruments used (Table 1). After removing heterogeneous genes and confounding genes, no possible heterogeneity was detected in 8 predicting risk factors (Table 1). No possible pleiotropy was detected in 8 predicting risk factors (Table 1). However, these associations remained consistent when outlier variants that were removed from the MRPRESSO analysis (Table 1). In the reverse MR Analysis, genetic liability to cervicalgia and LBP (*P* < 5E-6) was not associated with 8 modifiable risk factors (Table S1, Supplemental Digital Content, http://links.lww.com/MD/I937).

### 3.2. Effects of BMI and WC on potential mediators

**Figure 3.**
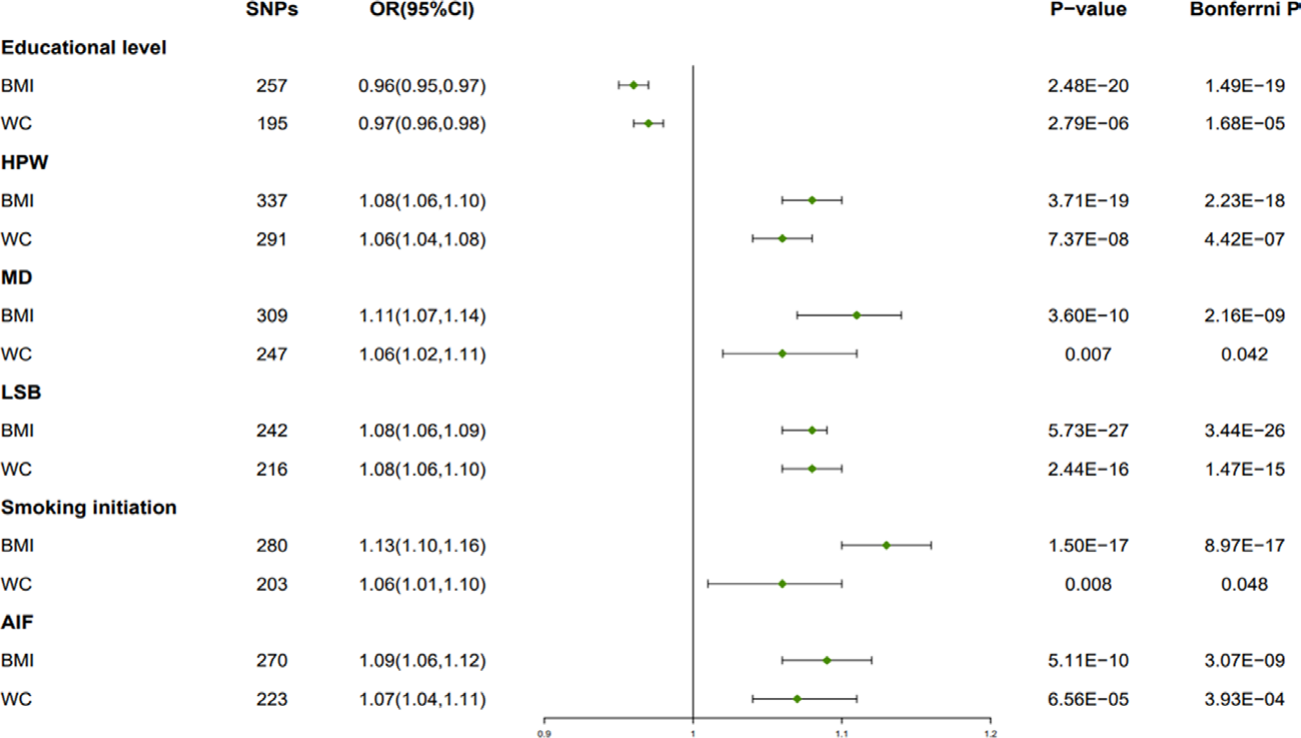
MR Analysis of BMI and WC with mediating factors. AIF = alcohol intake frequency, BMI = body mass index, HPW = heavy physical work, LSB = leisure sedentary behavior, MD = major depression, MR = Mendelian randomization, WC = waist circumference.

In the UVMR analysis of BMI and WC, it was found that BMI and WC had significant causal effect on mediating factors (Fig. 3). BMI (OR = 0.96, 95% CI: 0.95–0.97, *P* = 2.48E − 20, Bonferrni *P* = 1.49E − 19) and WC (OR = 0.97, 95% CI: 0.96–0.98, *P* = 2.79E − 06, Bonferrni *P* = 1.68E − 05) were negatively associated with the risk of educational level. BMI (OR = 1.08, 95% CI: 1.06–1.10, *P* = 3.71E − 19, Bonferrni *P* = 2.23E − 18) and WC (OR = 1.06, 95% CI: 1.04–1.08, *P* = 7.37E − 08, Bonferrni *P* = 4.42E − 07) was positively associated with the risk of HPW. BMI (OR = 1.11, 95% CI: 1.07–1.14, *P* = 3.60E − 10, Bonferrni *P* = 2.16E − 09) and WC (OR = 1.06, 95% CI: 1.02–1.11, *P* = .007, Bonferrni *P* = .042) was positively associated with the risk of MD. BMI (OR = 1.08, 95% CI: 1.06–1.09, *P* = 5.73E − 27, Bonferrni *P* = 3.44E − 26) and WC (OR = 1.08, 95% CI: 1.06–1.10, *P* = 2.44E − 16, Bonferrni *P* = 1.47E − 15) was positively associated with the risk of LSB. BMI (OR = 1.13, 95% CI: 1.10–1.16, *P* = 1.50E − 17, Bonferrni *P* = 8.97E − 17) and WC (OR = 1.06, 95% CI: 1.01–1.10, *P* = .008, Bonferrni *P* = .048) was positively associated with the risk of smoking initiation. BMI (OR = 1.09, 95% CI: 1.06–1.12, *P* = 5.11E − 10, Bonferrni *P* = 3.07E − 09) and WC (OR = 1.07, 95% CI: 1.04–1.11, *P* = 6.56E − 05, Bonferrni *P* = 3.93E − 04) was positively associated with the risk of AIF.

BMI and WC were negatively associated with educational level, but positively associated with HPW, MD, LSB, smoking initiation, and AIF (Fig. 3). The results of the above-mentioned MR analysis were robust to several sensitivity analyses (Table 2 and Table S2, Supplemental Digital Content, http://links.lww.com/MD/I938). All F statistics exceeded 10 for all instruments, indicating satisfactory strength of the genetic instruments used (Table 2). After removing heterogeneous genes and confounding genes, no possible heterogeneity was detected in 6 mediating factors (Table 2). No possible pleiotropy was detected in 6 mediating factors (Table 2). However, these associations remained consistent when outlier variants that were removed from the MRPRESSO analysis (Table 2).

### 3.3. Mediating effects of mediators in the association between BMI/WC and cervicalgia/LBP

**Figure 4.**
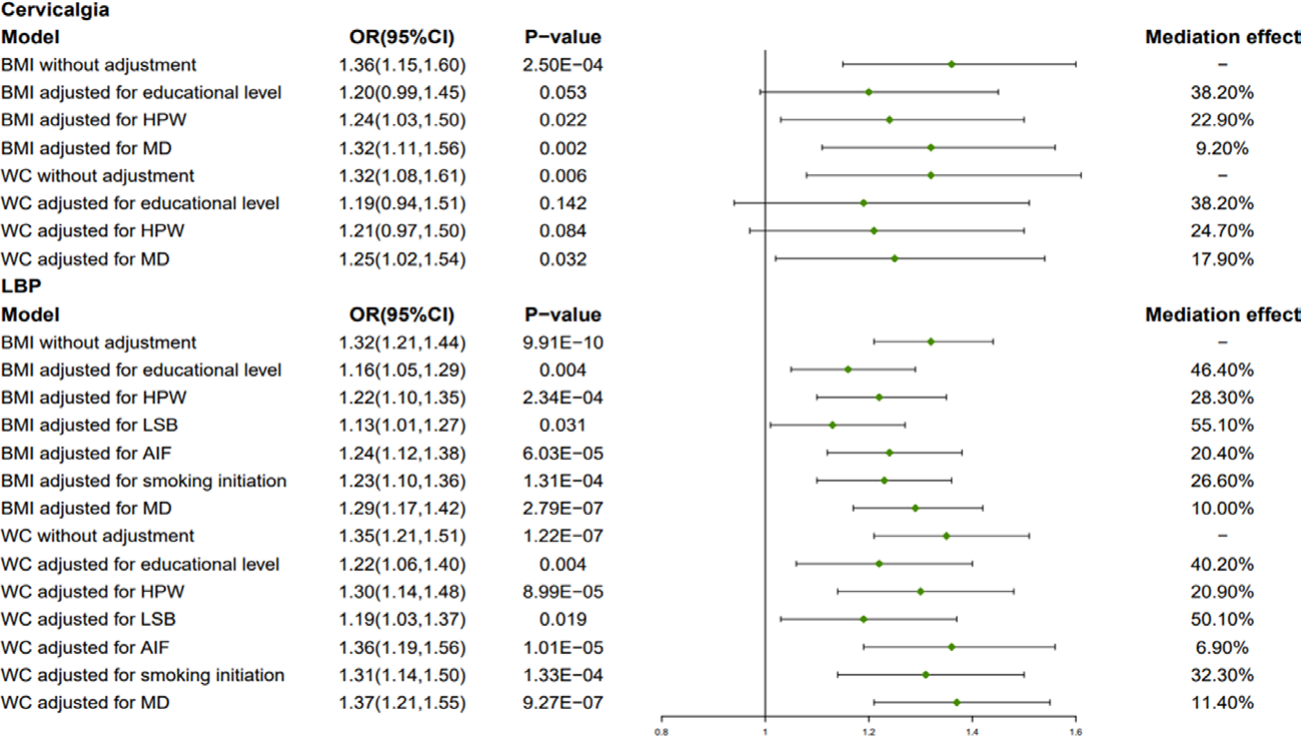
Mediation MR Analysis of mediating factors with BMI and WC. AIF = alcohol intake frequency, BMI = body mass index, HPW = heavy physical work, LSB = leisure sedentary behavior, LBP = low back pain, MD = major depression, MR = Mendelian randomization, WC = waist circumference.

In the multivariate MR and mediation MR analysis, it was revealed that mediators significantly mediated the effect of BMI and WC on the risk of cervicalgia and LBP (Fig. 4 and Table S3, Supplemental Digital Content, http://links.lww.com/MD/I939). Ranked by mediated proportions of selected mediators, the largest causal mediator from BMI to cervicalgia was educational level (38.20%), followed by HPW (22.90%), and MD (9.20%); The largest causal mediator from WC to cervicalgia was educational level (38.20%), followed by HPW (24.70%), and MD (17.90%); However, the largest causal mediator from BMI to LBP was LSB (55.10%), followed by educational level (46.40%), HPW (28.30%), smoking initiation (26.60%), AIF (20.40%), and MD (10.00%); The largest causal mediator from WC to LBP was LSB (50.10%), followed by educational level (40.20%), smoking initiation (32.30%), HPW (20.90%), MD (11.40%), and AIF (6.90%).

## 4. Discussion

The present MR study indicated that genetic predisposition to HPW, WC, BMI, and MD was associated with the increased risk of cervicalgia. Similarly, genetic predisposition to HPW, AIF, sedentary behavior, WC, BMI, smoking initiation, and MD was associated with the increased risk of LBP. In addition, genetic predisposition to educational level was associated with the reduced risk of cervicalgia and LBP. Educational level, HPW, and MD mediated the effect of BMI and WC on the risk of cervicalgia. while educational level, HPW, AIF, LSB, smoking initiation, and MD mediated the effect of BMI and WC on the risk of LBP.

Previous studies have agreed on whether elevated BMI, particularly being overweight or obese, promotes the development of cervicalgia and LBP. Ozcan–Eksi et al^[24]^ have revealed that the severity of LBP at the L4 to L5 and L5 to S1 intervertebral disc levels was strongly associated with BMI. A cross-sectional study conducted by Samartzis et al^[25]^ showed that the number and severity of disc degeneration were significantly increased in obese individuals. Similarly, a meta-analysis involving 1748 patients with disc disease showed that obesity was a major risk factor for disc disease, compared with age and sex.^[26]^ Accumulating evidence has shown that obesity is the most important risk factor for disc disease.^[27,28]^ These findings are consistent with our observations using the MR method.

The prevalence of cervicalgia and LBP significantly varies among different occupational groups. The occurrence of cervicalgia and LBP is mainly caused by long-term work at the desk, fatigue, cold or trauma. In addition to the abnormal development of vertebral column, long sitting and poor posture are also the main pathogenic factors.^[29]^ In the observational research on forklift operators,^[30]^ helicopter pilots,^[31]^ ship operators,^[31]^ and Swiss medical students,^[32]^ it was found that the incidence rate of cervicalgia and LBP was significantly higher than the local average incidence rate. Shahla et al^[33]^ demonstrated that the nature of work had a great correlation with cervicalgia and LBP through a 2-year follow-up of 264 staff. The present study revealed that the college or university degree was negatively correlated with the risk of cervicalgia and LBP, while the heavy manual or physical work was positively correlated with the risk of cervicalgia and LBP, which could be related to the nature of their work. Geertje et al^[34]^ pointed out that the occurrence of cervicalgia and LBP was related to the fixed posture and bending trunk for long-term work through a systematic evaluation. Bart et al systematic evaluation^[35]^ showed that those who frequently used computers were at the higher risk of cervicalgia and LBP. The present study revealed that there was a positive correlation between LSB and the risk of LBP. This may be because LSB is more likely to cause an increase in disc compression force^[36]^ and long-term overload of lumbar muscles, resulting in functional disorders, loss of stability, and changes in physiological radian of the entire lumbar vertebra, ultimately leading to LBP. Drinking and smoking are considered as risk factors for cervicalgia and LBP,^[3,37–40]^ and their mechanism may be attributed to the disturbance of intervertebral disc microcirculation. Palmer et al^[41]^ concentrated on the morbidity and occupational factors of 993 patients with neck pain in the UK, and confirmed that smoking has an inducing effect on cervicalgia. A cross-sectional study on 10,000 adults revealed a high correlation between smoking habits and the risk of cervicalgia and LBP, whereas did not find a correlation between AIF and the risk of cervicalgia.^[37]^ The present study indicated that smoking and AIF were positively correlated with the risk of LBP, while this finding was not generalizable to cervicalgia. This is inconsistent with the results of the above-mentioned research, and further research is needed to verify the findings of the present study and clarify the potential mechanism. Meanwhile, disc compression force is significantly higher in obese individuals.^[36]^ Studies have shown that microvascular rarefication and endothelial cell relaxation ability are significantly decreased in obese individuals,^[42]^ and the degree of microvascular dysfunction is positively correlated with the degree of obesity.^[43]^ This may be an important reason why obese individuals who enjoy LSB, smoking, and drinking are more likely to suffer from cervicalgia and LBP.

In observational studies, metabolic factors (e.g., type 2 diabetes,^[44–48]^ BMI,^[49–51]^ dyslipidemia,^[52,53]^ depression^[54,55]^) are associated with the risk of cervicalgia and LBP. However, it is elusive whether these metabolic factors have a causal relationship with the risk of cervicalgia and LBP, because most of previous studies were based on observational data. The present study revealed that BMI, WC, and MD were positively correlated with the risk of cervicalgia and LBP, but type 2 diabetes and lipids were not causally associated with risk for cervicalgia and LBP (Table S2, Supplemental Digital Content, http://links.lww.com/MD/I938). The results of this MR study are in partial consistent with those of the majority of previous observational studies. Mediating factors are mainly closely associated with overweight and obesity. Estimating the independent effects of these mediating factors has an important clinical significance.^[56]^ The results of the present study suggested that applying intervention to promote obese individuals lifestyles may be more beneficial to prevent cervicalgia and LBP. In addition, mediating MR analysis found that educational level, HPW, and MD mediated the effect of BMI and WC on the risk of cervicalgia. Educational level, HPW, AIF, sedentary behavior, smoking initiation, and MD mediated the effect of BMI and WC on the risk of LBP.

The main advantage of this study is that the implementation of MR method could reduce the interference of confounding factors with reverse causality to the results. To our knowledge, this is the first MR analysis on the causal association between obesity and cervicalgia and LBP, as well as the effect of possible mediating factors. Nevertheless, the study has some limitations. First, this study was limited to Europeans. Although it could reduce the bias caused by population stratification, it was not proved to be applicable to other races. Second, this study could not resolve the unobserved pleiotropy; hence, the results might be biased. Finally, for exposure factors, such as AIF and smoking, nonlinear association could not be estimated by MR analysis based on genetic statistics. Similarly, gene-environment interactions, age, and gender were not assessed in pooled data, and further research is therefore required.

## 5. Summary

This finding emphasizes that avoiding HPW and maintaining a stable mood in obese individuals may be an effective approach to prevent cervicalgia. Additionally, reducing LSB, avoiding HPW, quitting smoking and drinking, and maintaining a stable mood in obese individuals may be an effective approach to prevent LBP. The negative associations between educational level with the risk of cervicalgia and LBP need to be confirmed in the next studies.

## Acknowledgments

The authors acknowledge the participants and investigators of the UK Biobank and FinnGen studies.

## Author contributions

**Conceptualization:** Qiang Zheng.

**Data curation:** Qiang Zheng.

**Funding acquisition:** Qiang Zheng.

**Investigation:** Li Gou, Qiang Zheng.

**Project administration:** Li Gou.

**Software:** Li Gou.

**Writing – original draft:** Li Gou.

**Writing – review & editing:** Qiang Zheng.

## Supplementary Material






